# Injectable Thermosensitive Hyaluronic Acid Hydrogels for Chondrocyte Delivery in Cartilage Tissue Engineering

**DOI:** 10.3390/ph16091293

**Published:** 2023-09-13

**Authors:** Chih-Hao Chen, Hao-Hsi Kao, Yen-Chen Lee, Jyh-Ping Chen

**Affiliations:** 1 Department of Chemical and Materials Engineering, Chang Gung University, Kwei-San, Taoyuan 33302, Taiwan; 2 Department of Plastic and Reconstructive Surgery, Chang Gung Memorial Hospital at Keelung, Chang Gung University College of Medicine, Keelung 20401, Taiwan; 3 Division of Nephrology, Chang Gung Memorial Hospital at Keelung, Chang Gung University College of Medicine, Keelung 20401, Taiwan; 4 Department of Neurosurgery, Chang Gung Memorial Hospital at Linkou, Kwei-San, Taoyuan 33305, Taiwan; 5 Research Center for Food and Cosmetic Safety, College of Human Ecology, Chang Gung University of Science and Technology, Kwei-San, Taoyuan 33302, Taiwan; 6 Department of Materials Engineering, Ming Chi University of Technology, Tai-Shan, New Taipei City 24301, Taiwan

**Keywords:** hydrogel, chondrocytes, tissue engineering, thermosensitive, hyaluronic acid

## Abstract

In this study, we synthesize a hyaluronic acid-*g*-poly(N-isopropylacrylamide) (HPN) copolymer by grafting the amine-terminated poly(N-isopropylacrylamide) (PNIPAM-NH_2_) to hyaluronic acid (HA). The 5% PNIPAM-NH_2_ and HPN polymer solution is responsive to temperature changes with sol-to-gel phase transition temperatures around 32 °C. Compared with the PNIPAM-NH_2_ hydrogel, the HPN hydrogel shows higher water content and mechanical strength, as well as lower volume contraction, making it a better choice as a scaffold for chondrocyte delivery. From an in vitro cell culture, we see that cells can proliferate in an HPN hydrogel with full retention of cell viability and show the phenotypic morphology of chondrocytes. In the HPN hydrogel, chondrocytes demonstrate a differentiated phenotype with the upregulated expression of cartilage-specific genes and the enhanced secretion of extracellular matrix components, when compared with the monolayer culture on tissue culture polystyrene. In vivo studies confirm the ectopic cartilage formation when HPN was used as a cell delivery vehicle after implanting chondrocyte/HPN in nude mice subcutaneously, which is shown from a histological and gene expression analysis. Taken together, the HPN thermosensitive hydrogel will be a promising injectable scaffold with which to deliver chondrocytes in cartilage-tissue-engineering applications.

## 1. Introduction

As a form of hyaline cartilage, the articular cartilage consists of an extracellular matrix (ECM) sparsely populated with chondrocytes. However, the cartilage tissue shows very low metabolic activity from the limited cell proliferation. Furthermore, due to the low cellularity and lack of blood vessels with which to transport nutrients, the articular cartilage has a limited regeneration ability, and surgical intervention is required to repair the defective tissue [1]. Among the various methods with which to repair articular cartilage, the most recognized form of regenerative cell therapy is autologous cell transplantation, where autologous chondrocytes are transplanted after cell expansion in a monolayer cell culture in vitro. However, chondrocytes cultured in vitro usually show dedifferentiation during proliferation and lose their physiological properties and functions, which is currently a critical limitation when using expanded chondrocytes for cell transplantation [2]. As the conventional method for cartilage defect repair usually forms fibrocartilage tissues of inferior properties, cartilage tissue engineering emerges as an alternative for repairing the damaged tissue, by regenerating neo-cartilage at the defect site. Although tissue engineering can deliver cells in 3D scaffolds, the chondrocytes still need to be cultured in a monolayer culture for cell expansion, during which the dedifferentiation of chondrocytes usually occurs with increased cellular spreading [3]. Many cell culture methods attempt to solve this limitation by regulating the redifferentiation of chondrocytes in vitro, including the creation of a suitable 3D microenvironment with which to modulate the differentiated phenotype of the chondrocytes [4].

Chondrocytes are prone to dedifferentiation during the 2D culture process in tissue culture polystyrene (TCPS) through the transition to a fibroblastic phenotype. The dedifferentiated chondrocytes will produce less cartilage ECM, and the newly formed neo-cartilage will have impaired mechanical properties in vivo [5]. Although growth factor supplements may promote the redifferentiation of the expanded chondrocytes, the maintenance of the chondrogenic phenotype during the chondrocyte culture is still a challenging task [6]. Alternatively, a 3D scaffold for cartilage tissue engineering may maintain the phenotype or even cause the redifferentiation of chondrocytes [7]. Therefore, a tissue-engineering approach using chondrocytes to regenerate damaged cartilage tissues by combining injectable biodegradable scaffolds with chondrocytes for cell delivery looks attractive. However, the ideal scaffold for cell delivery in cartilage regeneration should preferably provide injectable properties for the non-invasive delivery of the cell/scaffold constructs, as well as provide a suitable milieu with which to maintain the phenotype of the chondrocytes for implantation [8]. Specifically, cell delivery using an injectable hydrogel may serve these purposes [9].

With their high water content and similarity to the structure of a native ECM, hydrogels could be employed as scaffolds in tissue-engineering applications [10]. Specifically, with their good biocompatibility and mechanical properties similar to soft tissues, hydrogels can be used in cartilage tissue engineering [11]. For a minimally invasive delivery, hydrogels can act as cell carriers for the regeneration of damaged cartilage tissues by delivering encapsulated chondrocytes [12]. Injectable hydrogels have been used clinically for local deformation in cartilage by mixing the patient’s expanded cells with the hydrogel, followed by injecting the mixture into the damaged area in the cartilage. This method provides a low level of invasiveness and the possibility of adjusting the implant to the irregular shape of the defect for cell delivery [13]. An example of an injectable hydrogel for cell delivery would be entrapping cells in a polymer solution showing sol-to-gel phase change behavior in situ. This is a feasible choice for cartilage tissue engineering using a non-invasive cell delivery approach [14]. However, the gelation process in the presence of cells for cell entrapment can be harmful to the encapsulated cells in the hydrogel scaffolds [15]. At room temperature, below the phase transition temperature, the thermoresponsive hydrogel polymer solution can mix with chondrocytes and the mixture can be delivered through a syringe needle to the body, after which sol-to-gel transition occurs and the in situ formed hydrogel can preserve high cell viability. Therefore, a thermosensitve hydrogel may deliver cells in a less invasive way and act as a cartilage-tissue-engineering scaffold.

Hyaluronic acid (HA) is a natural polysaccharide and a key component in the connective tissue ECM. It is abundant in the synovial fluid, showing a high water absorption rate and high water retention ability [16]. The high viscous and viscoelastic natures have made HA an excellent lubricant and shock absorber for the protection of joints [17]. It is also known to influence the behavior of chondrocytes from a sophisticated signaling pathway, which leads to the enhancement of the cellular functions of the chondrocytes [18]. Exogenous HA shows a direct biological effect and stimulates chondrocyte metabolism, which is believed to occur through interactions with CD44 receptors on the cell surface [19]. The thermosensitive polymer poly(N-isopropylacrylamide) (PNIPAM) exhibits a sol-to-gel phase transition in an aqueous solution. This polymer is water-soluble at temperatures below its lower critical temperature (LCST) around 30 °C, and forms a precipitate above this temperature [20]. An intelligent biopolymer system can be developed based on PNIPAAM [21]. By modifying the PNIMPPA with natural polymers, the LCST of the copolymers can be adjusted, which may also improve the mechanical strength and biocompatibility of the copolymer hydrogel for cell entrapment [22]. With the in situ sol-to-gel phase transition at physiological temperature, a PNIPAM-based copolymer can uniformly mix with cells at temperatures below its LCST and can be quickly injected into a desirable site in vivo, after which a physiological temperature above the LCST can induce gel formation and the retention of the delivered cells [23]. The formed hydrogel is expected to provide a cell-friendly 3D environment for cell proliferation and ECM secretion [24]. With regard to the minimally invasive cell delivery strategy using thermosensitive copolymer hydrogels, an injectable hydrogel is preferred over rigid scaffolds in treating irregularly shaped defects, and is a less time-consuming and more cost-effective method than open surgery [25].

A thermosensitive PNIPAM/HA copolymer was synthesized and conjugated with cell adhesive peptides [26]. The cell adhesive thermogelling copolymer can be used for the noninvasive delivery of a liquid suspension of cells into the delicate subretinal space in retinal therapeutics. A thermosensitive chondro-supportive hydrogel was synthesized by blending crosslinked HA with triblock copolymers of polyethylene glycol and partially methacrylated poly[N-(2-hydroxypropyl) methacrylamide mono/dilactate] (polyHPMA-lac-PEG) triblock copolymers [27]. The addition of HA to the triblock copolymer improved the printability of the hydrogel, which provides an attractive system for the design of a cell-laden construct for cartilage regeneration. A thermosensitive injectable hydrogel was synthesized by crosslinking HA with Pluronic F-127 in situ for the delivery of chondrocyte-derived exosomes, in order to suppress cartilage destruction in osteoarthritis [28]. Through an intra-articular injection, the exosome-incorporated hydrogel can significantly prevent cartilage destruction by promoting cartilage matrix formation through the controlled release of the exosomes at damaged cartilage sites. Thermo- and pH-sensitive glycosaminoglycan-based copolymers were synthesized by grafting PNIPAM to heparin and chondroitin sulfate [29]. These copolymers can prolong the release of dexamethasone phosphate after gelling and show no toxicity to the ocular cells, implicating their use for topical ocular drug delivery.

Previously, we synthesized a chitosan-*g*-poly(N-isopropylacrylamide) copolymer by grafting carboxyl-terminated PNIPAM (PNIPAM-COOH) to chitosan [30,31]. This is used successfully as an injectable cell delivery thermosensitive hydrogel [32]. As an HA-based hydrogel is preferred for chondrocyte delivery, we synthesize an injectable thermosensitive HA hydrogel in this study by grafting amine-terminated PNIPAM (PNIPAM-NH_2_) to HA to form hyaluronic acid-*g*-poly(N-isopropylacrylamide) (HPN). We postulate that HPN can be mixed with chondrocytes and injected through a needle to form a hydrogel matrix in situ at body temperature. We analyze the physicochemical properties and biological properties of this hydrogel from an in vitro culture of chondrocytes. Finally, chondrocytes were suspended in an HPN copolymer solution and injected into nude mice to form ectopic cartilage tissue subcutaneously, to test its potential as an injectable scaffold for cartilage tissue engineering.

## 2. Results and Discussion

### 2.1. Synthesis and Characterization of Hydrogels

**Figure 1:**
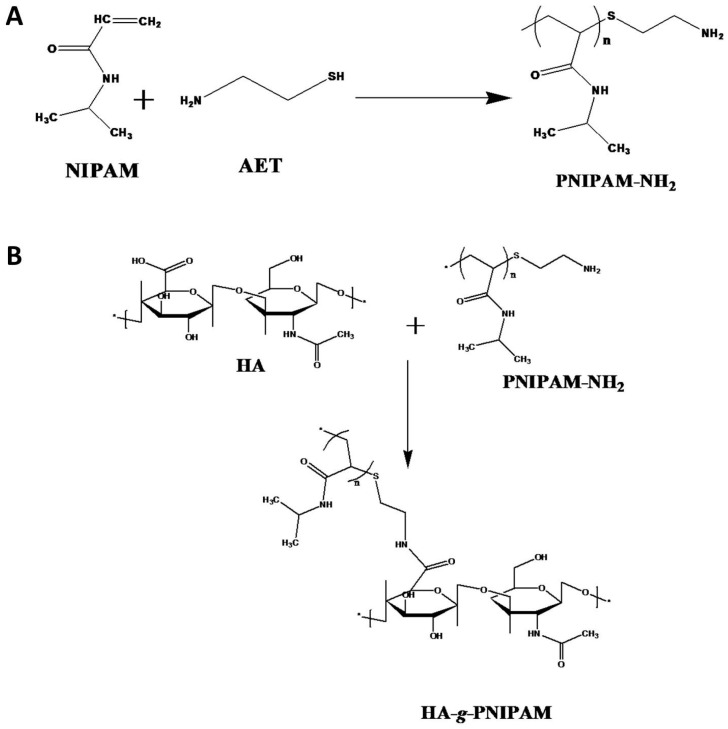
The synthesis of amine-terminated poly(N-isopropylacrylamide) (PNIPAM-NH_2_) using 2-aminoethanethiol (AET) as a chain transfer agent during free radical polymerization of N-isopropylacrylamide (NIPAM) (**A**), and the conjugation of PNIPAM-NH_2_ to HA to synthesize hyaluronic acid-*g*-poly(N-isopropylacrylamide (HPN) (**B**).

PNIPAM-NH_2_ was synthesized using 2-aminoethanethio (AET) as a chain transfer agent during the free radical polymerization of NIPAM (Figure 1A). The AET can terminate the polymerization, leaving a single amino group at the end of the PNIPAM-NH_2_ polymer. Using the 2,4,6-trinitrobenzene sulfonic acid (TNBSA) method to quantify the contents of the –NH_2_ end group in each PNIPAM-NH_2_ polymer chain, we can calculate the average molecular weight of PNIPAM-NH_2_ to be 22,000 Da. It should be noted that the molecular weight of PNIPAM-NH_2_ was determined from chain-end titration and it may be different from those determined from gel permeation chromatography (GPC), light scattering, or viscosity [33]. The GPC was also used to determine the molecular weight of PNIPAM-NH_2_ (Figure S1, Supplementary Materials), which agrees with that determined from end-group titration. By conjugating the end amine group in PNIPAM-NH_2_ to the carboxyl group in HA with 1-ethyl-3-(3-dimethylaminopropyl) carbodiimide (EDC) and N-hydroxysuccinimide (NHS), we can synthesize HPN (Figure 1B). The carboxyl groups in HA and the amine groups in PNIPAM-NH_2_ can form an amide bond after EDC activates the carboxyl groups, and the active intermediates can be stabilized by NHS.

The properties of hyaluronic acid-*g*-poly(N-isopropylacrylamide (HPN) was shown in Table 1. After the grafting reaction, the HPN was purified by thermally induced precipitation to remove free HA in the supernatant, followed by dialysis to remove PNIPAM-NH_2_, which has a much lower molecular weight than HPN. The presence of residual PNIPAM-NH_2_ in HPN has been checked by the TNBSA method, which can only detect primary amine groups. The result indicates that close to zero –NH_2_ groups were found in HPN. The –NH_2_ group is only present in PNIPAM-NH_2_ but not in HA and HPN. This indicates that the synthesized HPN does not contain PNIPAM-NH_2_ and the PNIPAM has been successfully grafted to HA. The grafting efficiency was calculated based on the weight percentage of the initial PNIAM-NH_2_ grafted to HA. Using the average molecular weight of HA (1.3 × 10^6^ Da) and the molecular weight of the repeating unit in HA, we calculated the available carboxyl groups in each HA molecule for conjugation with PNIPAM-NH_2_. By determining the weight of HA before and the weight of HPN after the grafting reaction, we can calculate the number of PNIPAM-NH_2_ chains grafted onto an HA molecule (grafting ratio). From the number of carboxyl groups per HA molecule and the grafting ratio, we can calculate the degree of grafting, which is the percentage of carboxyl groups in an HA molecule that has been conjugated with PNIPAM-NH_2_. The molecular weight of HPN was calculated based on the molecular weight of HA and the weight of the grafted PNIPAM chains as determined from the grafting ratio.

**Figure 2:**
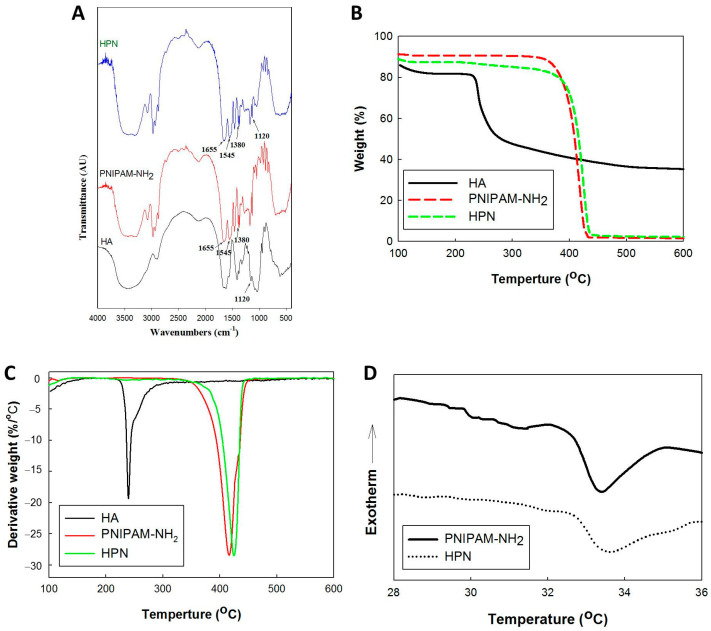
The Fourier-transform infrared (FTIR) spectra (**A**), thermogravimetric analysis (TGA) (**B**), differential thermal analysis (DTA) (**C**), and differential scanning calorimetry (DSC) thermograms (**D**) of amine-terminated poly(N-isopropylacrylamide) (PNIPAM-NH_2_, hyaluronic acid (HA), and hyaluronic acid-*g*-poly(N-isopropylacrylamide (HPN)).

The Fourier-transform infrared (FTIR) spectra of PNIPAM-NH_2_, HA, and HPN are shown in Figure 2A. The spectrum of PNIPAM-NH_2_ shows characteristic peaks at 1655 cm^−1^ from C=O stretching (amide I), and at 1545 cm^−1^ from N–H bending vibrations (amide II). The 1380 cm^−1^ peak represents the absorbance of methyl groups in the isopropyl groups in PNIPAM-NH_2_. The peak at 1120 cm^−1^ was from C–O–C stretching in HA. For HPN, the absorption peaks from amide bonds and the isopropyl groups in PNIPAM-NH_2_ could be identified, as well as the C–O–C peak in HA. This supports the successful conjugation of PNIPAM-NH_2_ to HA for HPN synthesis.

From the thermogravimetric analysis (TGA) in Figure 2B, the weight loss starts at ~200 °C for HA and ~400 °C for PNIPAM-NH_2_. The peak decomposition temperatures corresponding to the fastest weight loss rate are 237 °C and 418 °C for HA and PNIPAM-NH_2_, respectively, from the differential thermal analysis (DTA) curves (Figure 2C). For PNIPAM-NH_2_, a ~100% weight loss at 600 °C was found for this synthetic polymer, in contrast to ~40% for the natural polymer HA. The weight loss for HPN starts with PNIPAM-NH_2_ at ~400 °C, but the peak temperature shifts slightly to 424 °C, and a very small decomposition peak from HA is also detected. The residual weight of HPN is also similar to that of PNIPAM-NH_2_, as PNIPAM represents a >90% weight ratio in the HPN copolymer. Indeed, the weight percent of HA in HPN was calculated to be 6.1% from the molecular weight of PNIPAM-NH_2_ and the grafting ratio of HPN. Differential scanning calorimetry (DSC) was used to study the phase transition behavior of solutions of PNIPAM-NH_2_ and HPN (Figure 2D) [34]. All calorimetric profiles reveal an endothermic peak during the sol-to-gel phase transition, which is caused by the breaking up of the hydrogen bonds in the PNIPAM chains [35]. The polymer chains start to entangle from 32.7 °C for both samples. The peak endothermic temperatures are 33.3 °C and 33.5 °C for PNIPAM-NH_2_ and HPN, respectively, suggesting that HPN preserves the thermosensitive characteristics of PNIPAM-NH_2_. The enthalpies of phase separation were determined as the area under the corresponding endothermic peaks, which are 1.00 J/g and 0.89 J/g for PNIPAM-NH_2_ and HPN, respectively, indicating that the desolvation of PNIPAM-NH_2_ and HPN are different and require different amounts of energy. With the ~6% HA in HPN, the shift of peak temperature is marginal, and the more sensitive enthalpy change from the DSC analysis can better explain the desolvation. The enthalpy of phase separation, calculated from the area under the peak normalized by the sample mass, decreases for HPN compared with PNIPAM-NH_2_, indicating that less energy is required to undergo the phase transition. This trend suggests that the endothermic phase transition strongly depends on the presence of HA, which can hinder the contraction of the PNIPAM chains with a topological constraint [36]. The energy of the phase transition of HPN is influenced by the type of the constituting ionic groups in HA. The anionic carboxylic groups in the HPN copolymer structure can provide the ability for H-bonding with the NIPAM monomers randomly distributed along the copolymer chain. This can contribute to the disintegration of the thermosensitive hydration structure of PNIPAM-NH_2_.

**Figure 3:**
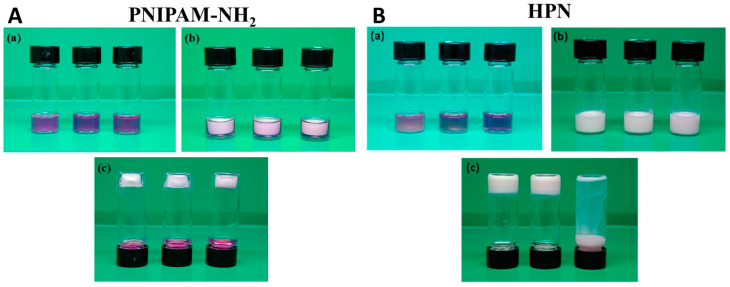
The phase transition behavior of amine-terminated poly(N-isopropylacrylamide) (PNIPAM-NH_2_) (**A**) and hyaluronic acid-*g*-poly(N-isopropylacrylamide (HPN) (**B**) at 10%, 5%, and 2.5% (*w*/*v*) concentrations from left to right. (**a**) Polymer solutions at room temperature (25 °C), (**b**) polymer solutions at 37 °C, and (**c**) sample bottles were inverted at 37 °C after polymer solutions were gelled. Trypan blue was added to the polymer solution for better visualization.

To study the gel formation, the phase transition kinetics of the PNIPAM-NH_2_ and HPN solution were studied at the 2.5%, 5%, and 10% polymer concentrations (Figure S2, Supplementary Materials). In general, hydrogels formed from a polymer solution with a higher polymer concentration show a faster response to the phase change induced by a temperature change to 37 °C. This may be related to the higher heat transfer rate when a more concentrated polymer solution is used in the study. Nonetheless, the phase transition is completed in less than 300 s for all polymer solutions, indicating that the polymer solutions prepared at all polymer concentrations under study are suitable for entrapping cells through an in situ temperature change above its lower critical solution temperature. The gel formation ability of PNIPAM-NH_2_ and HPN were also observed at different polymer concentrations. As shown in Figure 3, PNIPAM-NH_2_ forms a stable hydrogel irrespective of the polymer concentration, as the formed hydrogel can attach to the bottom of the vial when the vial is inverted. However, HPN only forms a stable hydrogel when the polymer concentration is above 5%, as the hydrogel formed from the 2.5% polymer concentration is not complete and tends to detach from the bottom of the vial when the vial is inverted. Furthermore, the water contents in the PNIPAM-NH_2_ and HPN hydrogels at 37 °C were determined (Figure S3, Supplementary Materials). The water content in HPN is higher than that in PNIPAM-NH_2_ at comparable polymer concentrations, owing to the contribution from HA, which can bind more water. This implies that the diffusion of solutes through the interior of the hydrogel can be more facile in HPN. The water content also decreases with the polymer concentration due to the effect of the volume repulsion between the polymer molecules and water molecules. Hydrogels with a higher water absorption ability can provide a higher cell viability, as a higher water content will impose a lower resistance to the diffusion of solutes, nutrients, and oxygen in the hydrogel, and are beneficial for entrapped cells [37]. At the lowest polymer concentration (2.5%) under study, the HPN polymer solution cannot form a stable gel at 37 °C (Figure 3). The next higher concentration of HPN polymer solution (5%), which exhibits a higher water content than 10% HPN, was thus chosen for both polymers and subjected to a detailed gel formation analysis.

**Figure 4:**
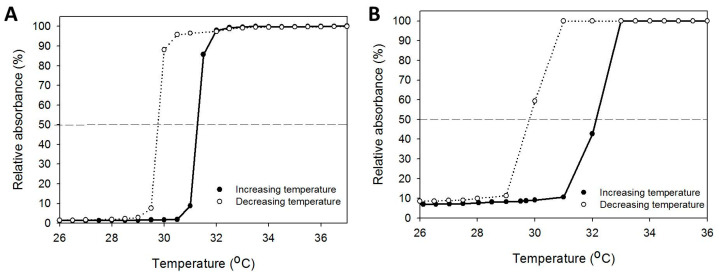
The phase transition behavior of PIPAM-NH_2_ (**A**) and HPN (**B**) by measuring the solution absorbance of a 5% (*w*/*v*) polymer solution at 470 nm during the heating/cooling cycle. The lines at 50% relative absorbance are used to determine the lower critical solution temperature.

To determine the LCST, a 5% polymer solution was subjected to a heating/cooling cycle, and the absorbance change with the temperature was recorded. As shown in Figure 4, the reversible soluble/insoluble characteristic was found for both thermosensitive polymers, indicating the sol and gel states can be changed reversibly [38]. The absorbance increases from the sol-to-gel phase transition and the absorbance decreases from the gel-to-sol phase transition at around 30 °C. However, a hysteresis loop exists during the heating/cooling cycle from the resistance to the disintegration of the entangled polymeric molecular chains in the hydrogel network [39]. The LCST is found when the solution turbidity is half of the maximum value in the heating and cooling curves. HPN has a slightly higher LCST (32.4 °C) than PNIPAM-NH_2_ (31.3 °C) during heating, but it showed a similar LCST with PNIPAM-NH_2_ (29.7 °C) during cooling. This slight shift in the LCST may be due to the hydrophilic hydroxyl (–OH) and carboxylic acid (–COOH) groups in HA, which increases the LCST during gel formation [40].

**Figure 5:**
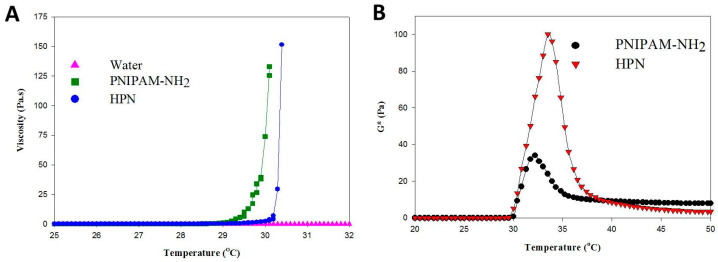
The viscosity (**A**) and complex shear modulus (**B**) of 5% (*w*/*v*) PNIPAM-NH_2_ or HPN solution as a function of temperature.

Using water as a control, the change of polymer solution viscosity with temperature is shown in Figure 5A. At temperatures below the phase transition temperature, the change in solution viscosity is negligible and the polymer chains in the polymer solution have high mobility. A transition behavior separating the viscosity dependence on temperature for the polymer could be observed with the solution viscosity around the transition temperature suddenly starting to increase to mark the onset of the gelation process [41]. This abrupt viscosity increase represents the temperature when a polymer solution turns into a rigid hydrogel with the contribution from the PNIPAM chains. All polymer solutions show transition temperatures similar to the LCST as determined from solution absorbance, with the gelling temperature of PNIPAAM-NH_2_ being lower than HPN. As an index of mechanical strength, the complex shear modulus (G*) of PNIPAM-NH_2_ and HPN were measured at different temperatures (Figure 5B). The G* value rises sharply after passing the gel-forming temperature (~32 °C) as the polymer solution starts to form a gel-like structure. The subsequent decrease of G* by further increasing the temperature may be due to the shrinkage of the polymer hydrogel. The introduction of HA could significantly improve the mechanical strength of PNIPAM-NH_2_, with the peak G* value increasing from 35 Pa for PNIPAM-NH_2_ to 100 Pa for HPN. The results from the G* measurements thus underline the benefits of using HPN over PNIPAM-NH_2_ as a cell delivery vehicle. The grafting of PNIPAM-NH_2_ to HA not only leads to the acquisition of the thermoresponsive characteristics of PNIPAM, but also improves the mechanical strength of the formed hydrogel. Nonetheless, the G* value of HPN is smaller than chitosan-*g*-poly(N-isopropylacrylamide) (495 Pa) [32] and chondroitin sulfate-g-poly(N-isopropylacrylamide) (194 Pa) [42], due to the different molecular structure between these carbohydrate polymers. As the PNIPAM hydrogel has insufficient mechanical strength, copolymerization with natural polymers has been suggested as a feasible means through which to improve its mechanical properties [43]. Undoubtedly, by providing a higher gel stiffness from its in situ stabilization ability, the HPN hydrogel is preferred over a pure PNIPAM hydrogel to better support tissue growth.

**Figure 6:**
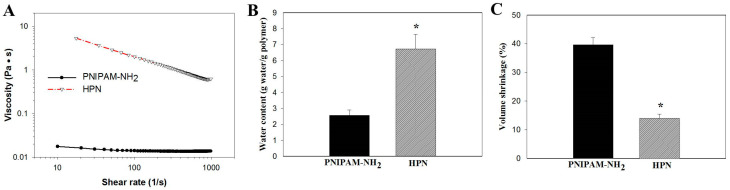
(**A**) The effect of shear rate on viscosity of 5% (*w*/*v*) PNIPAM-NH_2_ or HPN solution at 25 °C. The water content (**B**) and volume shrinkage (**C**) of 5% (*w*/*v*) PNIPAM-NH_2_ or HPN hydrogel at 37 °C. * *p* < 0.05 compared with PNIPAM-NH_2_.

For cell delivery, an injectable polymer hydrogel solution should show low resistance during shear flow, which demands the viscosity to decrease with the shear rate and allow the injection of the hydrogel. However, the hydrogel should also possess high viscosity after injection, with which the hydrogel can properly maintain its shape when at rest. To simulate the flow of the polymer solution through a syringe, the viscosity of the 5% (*w*/*v*) PNIPAM-NH_2_ or HPN solution at 25 °C was measured at different shear rates (Figure 6A). The PNIPAM-NH_2_ solution showed a Newtonian behavior with shear-rate-independent viscosity. The viscosity is very low compared with that of HPN, which shows non-Newtonian behavior with decreased viscosity at higher shear rates. By correlating the viscosity (μ) with the shear rate (γ) with the power law model as $\mu =K\gamma^{n-1}$, the power law index n value is 0.96 (coefficient of determination *r*^2^ = 0.998) and 0.45 (coefficient of determination *r*^2^ = 0.997) for PNIPAM-NH_2_ and HPN, respectively. The shear thinning of an HPN solution will meet the requirements for an injectable hydrogel formulation, where an injection through a syringe will be feasible by employing a higher shear rate for the flow of the polymer solution.

As the value of the water content in a hydrogel may influence the efficiency of the nutrient and waste transports within the hydrogel, the water content of 5% (*w*/*v*) PNIPAM-NH_2_ or HPN at 37 °C was measured. As shown in Figure 6B, the water content of HPN (6.7 ± 0.9 g water/g polymer) is 2.6 times that of PNIPAM-NH_2_ (2.6 ± 0.3 g water/g polymer). This could be attributed to the high water sorption and retention ability of HA, which results in HPN containing significantly more water than PNIPAM-NH_2_. This implies HPN can facilitate solute diffusion in its internal channels and HA may play an important role in stabilizing the structure integrity of polymer hydrogel networks. This point is supported after measuring the volume shrinkage of a 5% (*w*/*v*) PNIPAM-NH_2_ or HPN polymer solution after gelling.

The volume shrinkage percentage (%) is determined from the percentage of the water volume change after gel formation, and calculated from Equation (1):

(1) $$Volume\;shrinkage(\%)=-\frac{V_{w}}{V_{i}}\times 100$$

where V_w_ is the volume of water squeezed out from the hydrogel after gel formation at 37 °C and V_i_ is the volume of the initial polymer solution at 25 °C. As shown in Figure 6C, PNIPAM-NH_2_ showed considerable volume shrinkage (39.6%) after the phase change. In contrast, the addition of HA significantly decreased the extent of the collapse, and the volume shrinkage of HPN (14.0%) is only one-third that of PNIPAM-NH_2_. As a tissue-engineering scaffold, it is perceivable that substantial volume shrinkage during the sol-to-gel phase transition at physiological temperature for a hydrogel may damage the entrapped cells, squeeze cells out from the hydrogel, or hinder tissue repair with gaps existing between the implanted hydrogel and the surrounding tissues [44]. Overall, HPN is deemed as the preferred scaffold for cell delivery and will be employed for in vitro and in vivo studies.

### 2.2. In Vitro Studies

**Figure 7:**
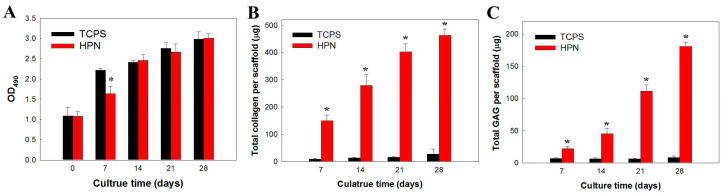
The cell proliferation (**A**), total collagen content (**B**), and total GAG content (**C**) when chondrocytes were cultured in 5% HPN hydrogel and on TCPS. * *p* < 0.05 compared with TCPS.

The proliferation of chondrocytes in HPN is shown in Figure 7A using methyl tetrazolium salt (MTS) assays to compare viable cell numbers from the solution absorbance at 490 nm (OD_490_). Chondrocytes seeded on TCPS were used as a control. The cell proliferates smoothly in HPN with a significantly lower cell number compared with the control on day 7. However, a similar cell number was found between the two groups after 14 days. A previous report indicates that the initial cell proliferation rate may be reduced during chondrocyte redifferentiation in a 3D scaffold after an expansion in a monolayer culture, and a higher cell proliferation rate is associated with low secretion rates of the cartilage-specific ECM components [45]. Collagen and glycosaminoglycans (GAGs) are two major ECM components found in cartilage, and their production by chondrocytes can be used as an index with which to assess cell redifferentiation. The GAGs are linear polysaccharides with disaccharide building blocks of amino sugars and uronic acid, and one or more GAG chains can bind to a core protein to form proteoglycans. Upregulating collagen production can provide increased mechanical stability by preventing GAG loss [46]. The total collagen (Figure 7B) and GAG (Figure 7C) content increased in the hydrogel scaffold with a culture time of up to 4 weeks when compared with the TCPS control, indicating the progressive accumulation of these cartilage ECM components. After 28 days, the amount of collagen and GAGs in HPN is 17-fold and 21-fold of those produced when chondrocytes are cultured on TCPS, respectively. These observations implied that HPN can provide a suitable milieu for chondrocyte redifferentiation, judging from the drastic increase of ECM production vs. the monolayer culture on TCPS.

**Figure 8:**
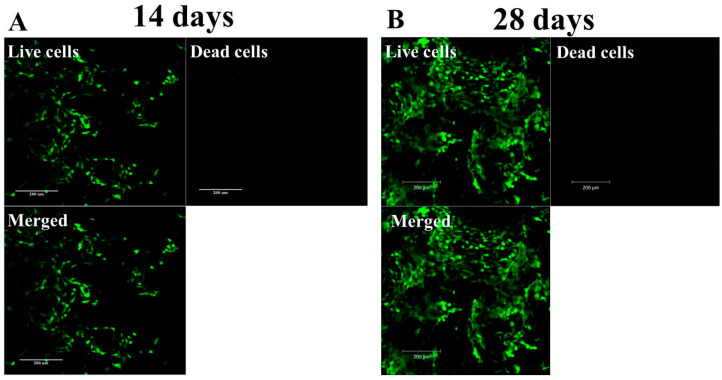
The viability of chondrocytes after cultured in HPN hydrogel for 14 days (**A**) and 28 days (**B**) with Live/Dead staining of cell/scaffold construct and confocal microscopy observation (bar = 200 μm).

To confirm the results of the increasing viable cell number and determine the cell distribution when chondrocytes are cultured in HPN, we carried out Live/Dead cell staining, followed by confocal microscopy observation. As shown in Figure 8, which is the Z-stack on a confocal microscope, the Live/Dead staining endorses the high cell viability of chondrocytes in the HPN hydrogel, where minimal dead cells and abundant viable cells showing green fluorescence were found in the hydrogel. The increasing number of viable cells from day 14 to day 28 is also consistent with those determined from the MTS assays.

**Figure 9:**
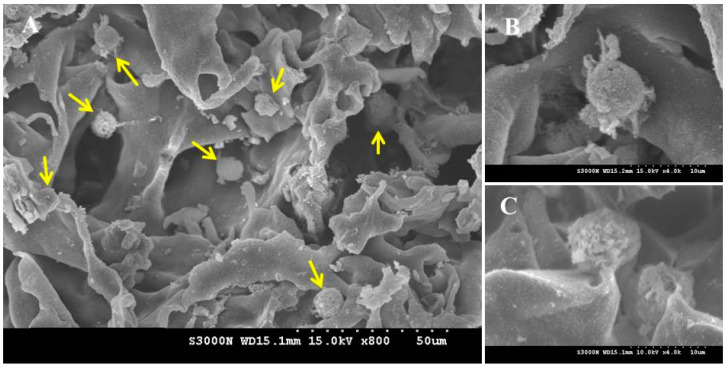
The scanning electron micrographs of chondrocytes after being cultured for 28 days in HPN hydrogel scaffold. (**A**) Magnification ×800, bar = 50 μm; (**B**,**C**) magnification ×4000, bar = 10 μm. The yellow arrows represent round chondrocytes found in (**A**).

The SEM image in Figure 9 indicates chondrocytes cultured in HA–CPN for 28 days were embedded within the secreted ECM, and the cells can retain the correct phenotypes. Instead of an elongated morphology on TCPS due to the dedifferentiation of the chondrocytes, chondrocytes in HPN maintain a spherical morphology, which is one of the characteristics of differentiated chondrocytes [47]. The dedifferentiation of chondrocytes is common after an in vitro cell culture on TCPS, where chondrocytes tend to lose the original morphology and show a diffused actin network with pronounced stress fiber formation [47]. Inducing the redifferentiation of chondrocytes by reversing the dedifferentiated phenotype is suggested from the spherical cell morphology of chondrocytes cultured in HPN. Overall, chondrocytes show a spherical cell morphology and produce ECM components after being cultured in the HPN hydrogel, indicating it can help to provide a milieu for maintaining a differentiated phenotype of entrapped chondrocytes for tissue-engineering applications.

**Figure 10:**
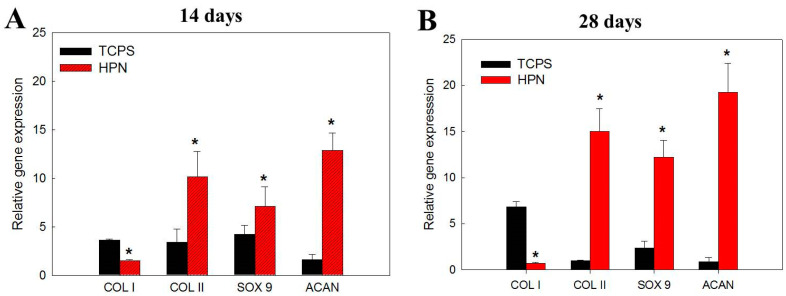
The gene expression analysis of type I collagen (COL I), type II collagen (COL II), SRY-box transcription factor 9 (SOX 9), and aggrecan (ACAN) by quantitative real-time polymerase chain reaction (qRT-PCR) after culture chondrocytes in HPN or on TCPS for 14 days (**A**) and 28 days (**B**). * *p* < 0.05 compared with TCPS.

Using a quantitative real-time polymerase chain reaction (qRT-PCR), we analyze the gene expression related to the chondrocyte phenotype, including the chondrogenic makers type II collagen (COL II), SRY-box transcription factor 9 (SOX 9), and aggrecan (ACAN), and the dedifferentiation marker type I collagen (COL I). From a differentiation pathway analysis, chondrocytes can secrete COL II, ACAN, and SOX 9 [48]. During the cell expansion on TCPS, dedifferentiated chondrocytes will show the upregulated gene expression of COL I, a protein characteristic of fibrotic tissues [49]. After the culture in HPN, the gene expression level of COL I was downregulated with the culture times, while it is upregulated for chondrocytes cultured on TCPS (Figure 10). Conversely, the gene expression level of chondrogenic genes COL II, ACAN, and SOX 9 were only upregulated in HPN and they showed a downregulated gene expression with the times when cultured on TCPS (Figure 10). A vast number of chondrocytes are usually required for cell delivery by the expansion of chondrocytes ex vivo after their isolation from a small cartilage specimen. However, evidence showed that this process usually results in altered gene expression profiles in passaged chondrocytes [50]. A more fibroblastic cell morphology, decreased COL II and ACAN expression, as well as increased COL I expression are commonly found during this dedifferentiation process. Dedifferentiated chondrocytes in the scaffolds usually form fibrocartilage neo-tissue, which cannot effectively repair the cartilage to fully restore its functions. By using HPN as a scaffold for the chondrocyte culture, the delivery of chondrocytes with an enhanced differentiated phenotype could be accomplished. This is due to the suitable 3D environment provided by HPN, which can induce the redifferentiation of chondrocytes from the dedifferentiated state experienced during the 2D culture process.

### 2.3. In Vivo Studies

**Figure 11:**
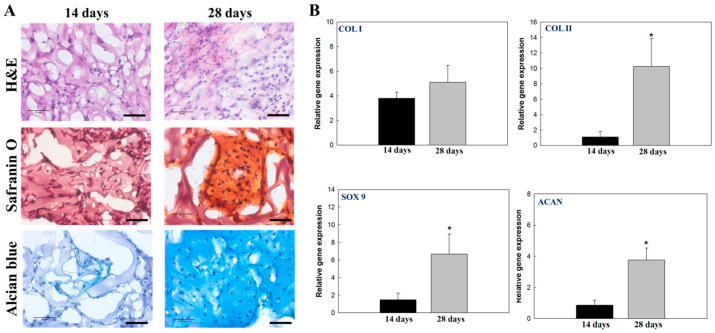
The hematoxylin and eosin (H&E), Safranin O, and Alcian blue staining ((**A**), bar = 50 μm), and the gene expression analysis of type I collagen (COL I), type II collagen (COL II), SRY-box transcription factor 9 (SOX 9), and aggrecan (ACAN) by quantitative real-time polymerase chain reaction (qRT-PCR) (**B**) of chondrocyte/hydrogel constructs 14- and 28-days post-implantation. * *p* < 0.05 compared with TCPS.

Taking advantage of the injectable feature of HPN, an HPN polymer solution was subcutaneously implanted in the flank of nude mice after mixing with chondrocytes for ectopic cartilage formation. As shown in Figure 11A, the H&E staining images indicate the subcutaneously implanted HPN in nude mice can provide an ectopic environment for the growth of chondrocytes, with the transplanted chondrocytes proliferating and showing an increased cell population. The tissue slice was stained with Safranin O for proteoglycan detection, and with Alcian blue for the detection of acidic polysaccharides (e.g., GAG) in the cartilage. Neo-cartilage formation was identified from positive Safranin O or Alcian blue staining results 14 days post-implantation, and the staining intensity can increase progressively with time up to 28 days. This indicated the transplanted chondrocytes can show a differentiated chondrogenic phenotype in vivo and secretes cartilage ECM, which is composed primarily of networks of GAG-containing proteoglycans.

From the qRT-PCR analysis, the upregulated expression of COL II, SOX 9, and ACAN were found to be correlated with time, as seen from the in vitro studies, supporting the idea that implanted chondrocytes in HPN can also maintain the chondrogenic phenotype in vivo for the formation of neo-cartilage tissues (Figure 11B). However, the expression of COL I was not downregulated as expected from the in vitro experiments, which might be due to the secretion of this protein by fibroblasts attaching to the outside of the implanted hydrogel [51]. Overall, the animal experiment underlines the ability to deliver chondrocytes by injecting a chondrocyte/HPN solution into the subcutaneous tissues of nude mice, which can be gelled at physiological temperature. The delivered chondrocytes can grow and produce key cartilage-specific proteins in vivo. As mentioned before, the dedifferentiation of chondrocytes is a barrier in cartilage tissue engineering for cartilage repair. Thus, the thermosensitive HPN hydrogel provides an injectable scaffold for cartilage tissue engineering, by providing a cell-friendly milieu for the phenotypic development of seeded chondrocytes in vitro, as well as for the in vivo maintenance of chondrogenic activity during the development of a cartilage-like tissue.

## 3. Materials and Methods

### 3.1. Materials

The activating reagents for amide bond formation, 1-ethyl-3-(3-dimethylaminopropyl) carbodiimide (EDC), and N-hydroxysuccinimide (NHS) were supplied by Acros Organics (Geel, Belgium). N-isopropylacrylamide (NIPAM) and 2,2′-azobis(isobutyronitrile) (AIBN) obtained from Showa Chemical (Tokyo, Japan) were recrystallized from n-hexane and methanol, respectively. Hyaluronic acid (HA) (average molecular weight = 1.3 × 10^6^ Da) was purchased from Bloomage Freda Biopharm Co. (Jinan, China). Alcian blue, Safranin O, 2-morpholinoethane sulfonic acid (MES), 2-aminoethanethiol (AET), 2,4,6-trinitrobenzene sulfonic acid (TNBSA), and Dulbecco’s modified Eagle’s medium/nutrient mixture F-12 (DMEM-F12) were purchased from Sigma-Aldrich (St. Louis, MO, USA). Fetal bovine serums (FBS) and Live/Dead cell viability/cytotoxicity kit were obtained from Thermo Fisher Scientific (Waltham, MA, USA). For methyl tetrazolium salt (MTS) assays, the CellTiter96 AQueous One Solution Cell Proliferation Assay kit was obtained from Promega (Madison, WI, USA).

### 3.2. Preparation of PNIPAM-NH_2_ and HPN

To prepare amine-terminated PNIPAM (PNIPAM-NH_2_), 30 mmol of recrystallized NIPAM monomer, 3 mmol of 2-aminoethanethiol (AET), and 0.21 mmol of AIBN were dissolved in 250 mL of benzene. Free radical polymerization was carried out by stirring (300 rpm) at 60 °C for 24 h in a water bath. After evaporating the solvent, acetone was added to dissolve the precipitate, followed by diethyl ether to induce secondary precipitation. The precipitate was dried in a vacuum and dissolved in distilled deionized water (DDI water) for dialysis with a dialysis membrane with 3500 molecular weight cut-off (MWCO) for 7 days at 4 °C, and the purified PNIPAM-NH_2_ was freeze-dried for storage.

To synthesize hyaluronic acid-*g*-poly(N-isopropylacrylamide) (HPN) copolymer, 5 g of PNIPAM-NH_2_ and 0.25 g of HA were mixed in 100 mL of 0.1 M MES buffer (pH 6.5). After adding 1.2 g of EDC and 0.24 g of NHS, the solution was stirred (180 rpm) at 25 °C for 12 h to complete the conjugation reaction. The solution was adjusted to 0.6 M NaCl by adding 3 M NaCl solution in a 50 °C water bath and incubated for 30 min. After ionic strength-induced precipitation, the solution was centrifuged at 50 °C for 20 min. The precipitate was redissolved in 0.1 M MES buffer (pH 6.5) and this process was repeated three times to purify HPN. The final product was dialyzed for 7 days using a 50,000 MWCO dialysis membrane and freeze-dried for storage. The freeze-dried HPN powder was placed in a covered dish and sterilized by UV light at 100 μJ/cm^2^ for 12 h for all in vitro and in vivo studies.

### 3.3. Characterization of PNIPAM-NH_2_ and HPN

The molecular weight of PNIPAM-NH_2_ was determined by chain-end titration by reaction of the end primary amino groups with TNBSA [52]. A PNIPAM-NH_2_ solution (0.5 mL) was reacted with 0.01% TNBSA (0.25 mL) in 0.1 M sodium bicarbonate (pH 8.5) at 25 °C for 2 h, then 0.25 mL of 10% SDS and 0.125 mL of 1 N HCl were added. The solution absorbance was measured at 335 nm and a calibration curve was constructed from glucosamine.

The grafting efficiency of HPN was calculated from Equation (2):

(2) $$Grafting\;Efficiency\left(\%\right)=\frac{\left(W_{HPN}-W_{HA}\right)}{W_{PNIPAM}}\times 100$$

where W_HPN_, W_HA_, and W_PNIPAM_ denote the weights of HPN, HA, and PNIPAM-NH_2_, respectively, used in the reactions.

The grafting ratio was calculated from Equation (3):

(3) Grafting ratio=(WHPN−WHA)MWPNIPAMWHAMWHA

where MW_PNIPAM_ and MWHA denote the molecular weight of PNIPAM-NH_2_ and HA, respectively.

The degree of grafting was calculated from Equation (4):

(4) $$Degree\;of\;grafting\left(\%\right)=\frac{Grafting\;ratio}{3768}\times 100$$

where 3768 is the number of –COOH groups in an HA chain based on its molecular weight (1,300,000). Therefore, the grafting ratio represents the number of PNIPAM-NH_2_ chains grafted onto an HA molecule, the grafting efficiency indicates the percentage of PNIAM-NH_2_ grafted to HA based on its initial amount used in the reaction, and the degree of grafting provides the percentage of carboxyl groups in HA used for the grafting reaction or the number of PNIPAM groups in 100 glucuronic acid units in the HA molecule. The molecular weight of HPN (MW_HPN_) was calculated from Equation (5):

(5) $${MW}_{HPN}={MW}_{HA}+{MW}_{PNIPAM}\times grafting\;ratio$$

The LCST was determined from a 5% polymer solution prepared in DDI water by measuring solution absorbance at 470 nm using a UV/VIS spectrophotometer. A circulating water bath was used for temperature control and the absorbance was recorded at increasing temperature from 25 to 40 °C at a rate of 0.25 °C/min, followed by decreasing temperature from 40 to 25 °C at a rate of 0.04 °C/min. The polymer solution was equilibrated at each tested temperature for 3 min before measuring the absorbance to allow the solution reach temperature equilibrium. By plotting solution absorbance vs. temperature, precipitation and dissolution curves were obtained and the LCST of a polymer solution was obtained from the temperature where solution turbidity reached 50% of the maximum value.

The water content of hydrogel was determined by placing 1 mL of 5% (*w*/*v*) polymer solution prepared in DDI water in a 4 mL vial and incubated at 37 °C incubator for 2 h for gel formation. After adding 1 mL of DDI water to the vial to maintain wettability, the vial was incubated for another 2 days at 37 °C, followed by removing the supernatant and weighing the fully hydrated hydrogel. The water content was calculated by dividing the water content by the weight of the polymer in the hydrogel. The viscosity of polymer solutions at different temperatures was determined using a rheometer (Carri-Med CSL^2^ 100, TA Instruments, New Castle, DE, USA) from 25 to 33 °C at 2 °C/min. A 60 mm diameter cone-plate with 1° angle was used and the parameters are set at 52 μm gap, 2 Pa shear stress, and 10 min ramp duration. For determining the rheological properties of polymer solutions at 25 °C, a 60 mm diameter cone-plate with 2° angle was used, and the parameters are set at 52 μm gap, 10 min ramp duration, and shear rate from 0 to 1000 s^−1^. The oscillatory analysis of hydrogel samples was performed using the same rheometer by determining the complex shear modulus (G*). The temperature was continuously increased from 20 to 50 °C at 3 °C/min using a 40 mm flat plate, 52 μm gap, 1 Pa shear stress, and 0.5 Hz frequency.

The chemical structure of the polymer was determined by Fourier-transform infrared (FTIR) spectroscopy. The polymer powder was mixed with KBr and scanned from 400 to 4000 cm^−1^ wavelength using a Horiba FT-730 spectrometer (Tokyo, Japan). The thermogravimetric analysis (TGA) was performed with a 10 mg sample in a nitrogen atmosphere heated to 600 °C at a 10 °C/min heating rate, using a TGA2050 thermogravimetric analyzer (TA Instruments, New Castle, DE, USA). The differential scanning calorimetry (DSC) analysis was performed to measure the phase transition temperatures of the polymer solutions using 5% copolymer solution, using a DSC2010 differential scanning calorimeter under 30 mL/min nitrogen. The samples were placed in hermetic pans to eliminate the possibility of water evaporation, and were scanned with a scan rate of 1 °C/min to 40 °C against an empty reference pan. The phase separation temperatures were measured as the maximum of the derivative of the heating thermograms.

### 3.4. In Vitro Studies

Chondrocytes were obtained from New Zealand white rabbits by digesting knee articular cartilage with a collagenase solution. The Chang Gung University Institutional Animal Care and Use Committee approved all animal experiment protocols. The cells were cultured with 90% DMEM-F12 + 10% FBS as a cell culture medium in T75-flasks and passage 2 chondrocytes were used in the study. For cell culture on tissue culture polystyrene (TCPS), which was used as a monolayer culture control, 1 × 10^5^ cells were seeded to each well of a 24-well cell culture plate. For cell culture in the hydrogel, 0.2 mL of 5% (*w*/*v*) sterilized polymer solution containing 1 × 10^5^ cells were prepared at 20 °C and placed in each well of a 24-well cell culture plate. After forming solid-like hydrogels by incubating at 37 °C, 2 mL of culture medium was added to each well for cell culture in a CO_2_ incubator.

To determine cell proliferation, the MTS assay was used. The samples were washed with PBS and reacted with 50 µL of MTS solution at 37 °C for 3 h. The viable cell number was determined from solution absorbance at 490 nm (OD_490_) using a microplate reader. To determine GAG secreted by chondrocytes, the samples were digested with 1 mL of digesting solution containing 50 mg/mL papain, 150 mM sodium chloride, 55 mM sodium citrate, 5 mM EDTA, and 5 mM cysteine. After incubating at 60 °C for 24 h, the GAG content was determined by reacting with 1,9-dimethyl-methylene blue from solution absorbance at 525 nm. A standard curve prepared with chondroitin sulfate was used to calculate the content of GAG. The total collagen contents were determined from the hydroxyproline content after reacting hydrolyzed samples with 4-(dimethylamino)benzaldehyde. The absorbance was determined at 540 nm and collagen content was calculated by assuming the weight of collagen to be 8 times that of hydroxyproline. To observe cell morphology from scanning electron microscopy (SEM), cell-seeded hydrogels were fixed in 2.5% glutaraldehyde for 3 h, and dehydrated with 50%, 70%, 80%, 90%, and 95% ethanol for 15 min each. The sample was incubated in 100% ethanol for 30 min and dried in a critical point dryer for observation with an S3000N scanning electron microscope (Hitachi, Tokyo, Japan).

For Live/Dead staining, cell-seeded hydrogels were incubated with a Live/Dead viability/cytotoxicity kit for 15 min, after which live cells can show green fluorescence by binding with calcein AM and dead cells can show red fluorescence by staining with ethidium homodimer-1. The washed samples were observed using a confocal microscope (Leica, TCS SP8) at excitation 404 nm/emission 517 nm wavelengths for live cells and 528 nm excitation/617 nm emission wavelengths for dead cells. Optical slices of a sample were obtained to a depth of 100 μm and Z-stack images from confocal microcopy were shown. Standard protocols of cDNA synthesis and RNA isolation were employed when the quantitative real-time polymerase chain reaction (qRT-PCR) was used to analyze the gene expression of type I collagen I (COL I), type II collagen (COL II), SRY-box transcription factor 9 (SOX 9), and aggrecan (ACAN) with glyceraldehyde-3-phosphate dehydrogenase (GAPDH) as a control. A MiniOpticon real-time PCR system was used for the reaction, and relative gene expression is presented using the cycle threshold method after normalizing with gene expression on day 0.

### 3.5. In Vivo Studies

The Chang Gung University Institutional Animal Care and Use Committee approved all animal experiment protocols. The backs of male nude mice (BALB/c) were sterilized with 75% alcohol under anesthesia. For assessment of ectopic cartilage formation, a 0.4 mL sterilized 5% HPN polymer solution containing 2 × 10^5^ chondrocytes were injected into the subcutaneous pocket of each animal. Cartilage formation was analyzed by sacrificing the animals 14- and 28-day post-implantation. The retrieved hydrogel samples were fixed in 4% paraformaldehyde, immersed in optimal-cutting-temperature (OCT) cryo-embedding matrix, and sectioned into 10 μm tissue specimens by cryostat sectioning and subjected to hematoxylin and eosin (H&E), Alcian blue, and Safranin O staining. The samples were also subjected to qRT-PCR analysis for the in vivo gene expression of COL I, COL II, SOX 9, and ACAN.

### 3.6. Statistical Analysis

All data presented were from at least triplicate experiments and reported as mean ± standard deviation (SD). The one-way analysis of variance (ANOVA) analysis has been employed for statistical analyses between groups, and *p*-values less than 0.05 are considered statistically different.

## 4. Conclusions

In this study, we successfully prepared a hydrogel scaffold for cartilage regeneration. The thermosensitive HPN hydrogel is a suitable injectable cell carrier for chondrocytes. With a low volume shrinkage percentage and high water retention ability induced by temperature change, the HPN hydrogel provides a cell-friendly 3D environment for chondrocytes. The entrapped chondrocytes show a high cell viability and proliferate well in the hydrogel, facilitating the delivery of a cell/polymer mixture that is minimally invasive for chondrocyte delivery. The hydrogel provides a suitable milieu for the entrapped chondrocytes to show a differentiated phenotype. The cells can redifferentiate from the lost phenotype experienced during cell expansion in HPN, by upregulating the expression of cartilage-specific genes and enhancing the production of GAGs. Moreover, the in vivo study demonstrated the feasibility of delivering chondrocytes using HPN for ectopic cartilage formation by subcutaneously implanting the cell/scaffold construct. The biocompatibility and the ability to maintain the chondrogenic phenotype shown by HPN suggest its use as a cell delivery vehicle for transplanting chondrocytes in cartilage tissue engineering. In the future, different preparation conditions of HPN may be studied to generate different compositions of the grafted copolymer in order to achieve better physicochemical and biological properties.

## Figures and Tables

**Table 1:** Properties of hyaluronic acid-*g*-poly(N-isopropylacrylamide (HPN).

Sample	Grafting Efficiency	Grafting Ratio	Degree of Grafting	Molecular Weight
HPN	77%	912	24%	2.14 × 10^7^

## Data Availability

The data presented in this study are available upon request from the corresponding author.

## Supplementary Materials

The following are available online at https://www.mdpi.com/article/10.3390/ph16091293/s1: Figure S1: Molecular weight of PNIPAM-NH_2_ determined by gel permeation chromatography; Figure S2: The phase transition kinetics of PNIPAM-NH_2_ and HPN solutions; and Figure S3: The water content of PNIPAM-NH_2_ and HPN hydrogels at 37 °C.
