# Supplementary material for: Injectable Thermosensitive Hyaluronic Acid Hydrogels for Chondrocyte Delivery in Cartilage Tissue Engineering
Source: Pharmaceuticals (Basel). 2023 Sep 13;16(9):1293. doi: 10.3390/ph16091293 (PMC10535600; doi:10.3390/ph16091293)
Supplement: Supplementary file 1 [file pharmaceuticals-16-01293-s001.zip › pharmaceuticals-2560478-SI.pdf]

Supplementary Materials

Injectable Thermosensitive Hyaluronic Acid Hydrogels for Chondrocyte Delivery in Cartilage Tissue Engineering

Molecular weight of PNIPAM-NH_2 determined by gel permeation chromatography

The molecular weight and molecular weight distribution of PNIPAM-NH_2 were determined by gel permeation chromatography (GPC) using a GPC KF 804 column, a Jasco PU980 pump, and a Waters 2414 refractive index (RI) detector with tetrahydrofuran (THF) as the mobile phase. A calibration curve was generated with polystyrene standards. Figure S1 shows the GPC trace of PNIPAM-NH_2. The weight-average molecular weight (M_W) and number-average weight (M_n) from the GPC analysis are 20,300 Da and 8,200 Da, respectively, and the dispersity index is 2.48.

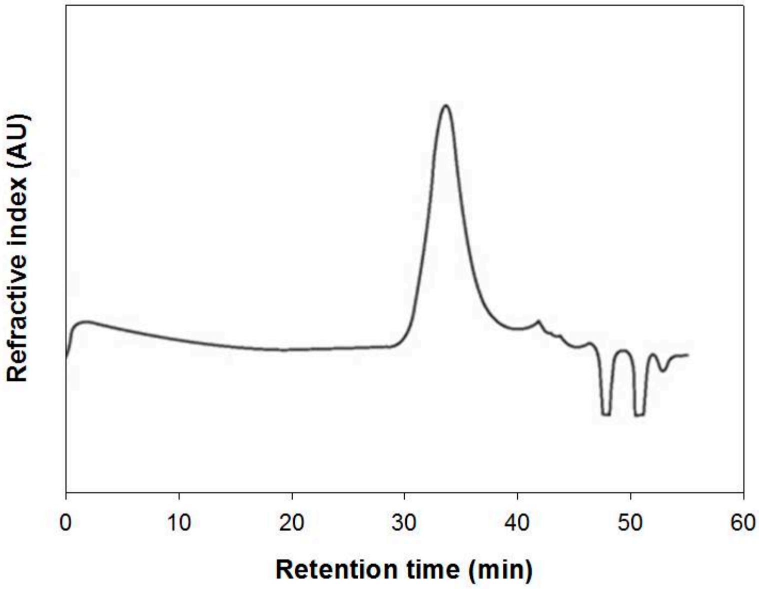

Figure S1. Gel permeation chromatography (GPC) trace of PNIPAM-NH_2 in THF.

Phase transition kinetics

The phase transition kinetics of the PNIPAM-NH_2 and HPN solution were studied with 2.5, 5, and 10% (w/v) of polymer aqueous solutions. The samples were equilibrated in a 25 °C incubator for 1 h, and then placed in an ultraviolet-visible (UV-Vis) spectrophotometer pre-equilibrated at 37 °C. The turbidity of the polymer solution was monitored by measuring the solution absorbance at 470 nm as a function of time. The gel formation time could be defined as the time at which the absorbance reached half of the maximum value.

As shown in Figure S2, the relative absorbance rose sharply as the surrounding temperature was shifted from 25 to 37 °C. The gel formation kinetics is generally slower at lower polymer concentrations. Hydrogels formed with a higher polymer concentration showed a faster

response to temperature change, leading to a resultant decrease in gel formation time, which may be related to the higher heat transfer rate of a more concentrated polymer solution. Nonetheless, the gel formation time is less than 5 min for all polymer solutions, indicating its suitability for entrapping cells through the phase change at temperatures above the lower critical solution temperature (LCST).

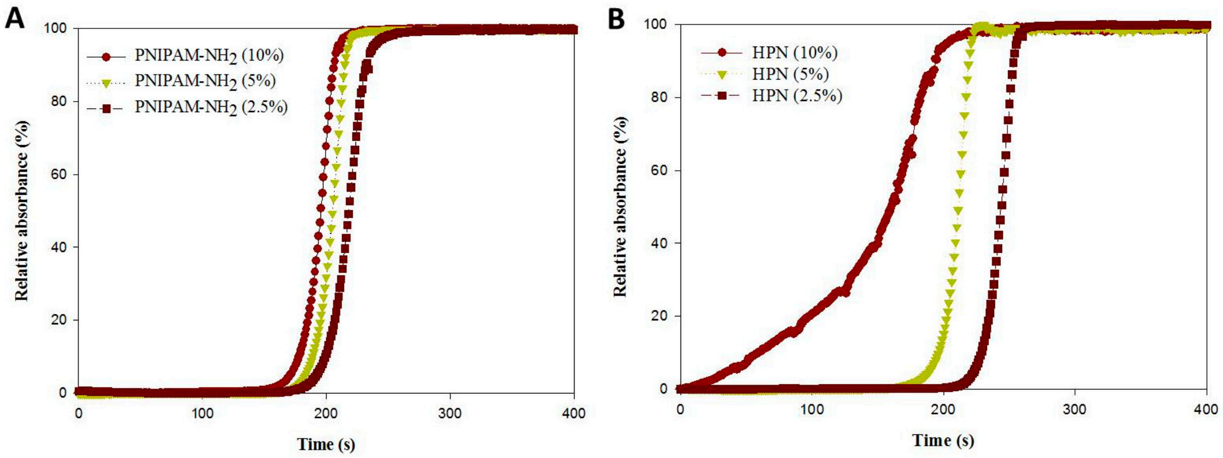

Figure S2. The phase transition kinetics of PNIPAM-NH_2 and HPN solutions. The solution absorbance of polymer concentration (2.5%, 5%, and 10% (w/v)) was measured at 470 nm when the temperature was suddenly changed from 25 °C to 37 °C.

Water content

To analyze the effect of a temperature change on the water content of the PNIPAM and HPN hydrogels, the water content of the polymer hydrogels was determined from the weight difference of the hydrogels before and after the phase transition. PNIPAM-NH_2 or HPN solutions of 2.5, 5, and 10% (w/v) concentrations were prepared in phosphate-buffered saline (PBS) and placed in a pre-weighted sample vial. The weight of the polymer solution was determined ($W_{polymer}$). After equilibrating the vials in a 37 °C water bath for 6 h to induce gel deswelling, the water squeezed out during gel formation was carefully removed using filter papers. The weight of the wet hydrogel was determined ($W_{hydrogel}$) for the calculation of the water content (%) from Equation 1.

$$\text{Water content (\%)} = \frac{W_{polymer} - W_{hydrogel}}{W_{polymer}} \times 100 \qquad (1)$$

As shown in Figure S3, the water content of the hydrogel equilibrated at 37 °C depends on the concentration and the types of hydrogels. Owing to the effect of the volume repulsion between the polymer molecules and water molecules, a more concentrated polymer solution resulted in hydrogels with lower water contents. The water content of HPN is higher than that of PNIPAM-NH_2 at a comparable polymer concentration, owing to the contribution of HA, which can bind more water.

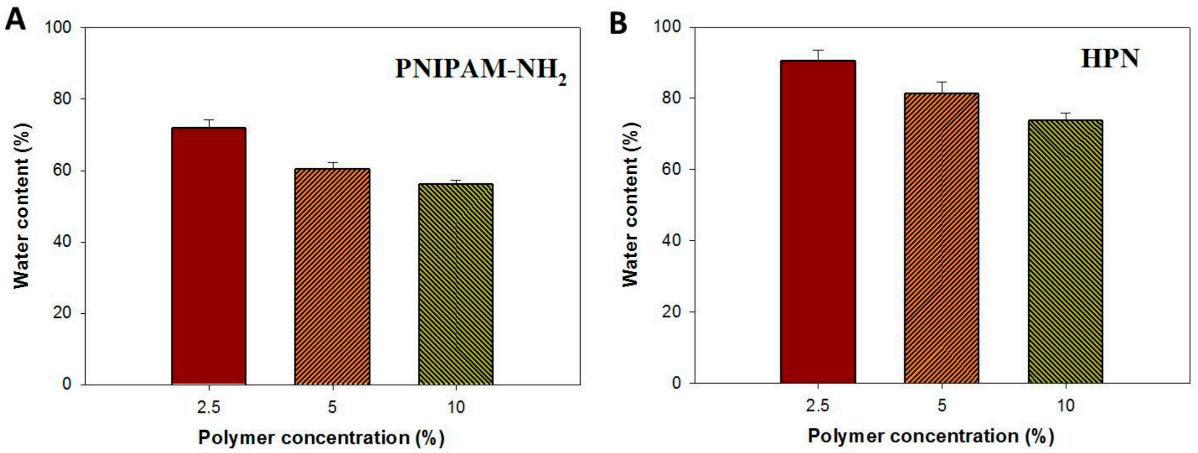

Figure S3. The water content of PNIPAM-NH_2 (A) and HPN (A) hydrogel at 37 °C prepared with 2.5%, 5%, and 10% (w/v) polymer concentration.
